# A Piercing Diagnosis – Occult Foreign Body as the Cause of Acute Inguinal Pain

**DOI:** 10.5811/cpcem.2020.12.50196

**Published:** 2021-01-26

**Authors:** Coral Bays-Muchmore, Deion T. Sims, Joel A. Gross, Jonathan S. Ilgen

**Affiliations:** * University of Washington School of Medicine, Seattle, Washington; † University of Washington School of Medicine, Department of Radiology, Seattle, Washington; ‡ University of Washington School of Medicine, Department of Emergency Medicine, Seattle, Washington

**Keywords:** Occult foreign body, inguinal pain

## Abstract

**Case Presentation:**

A 35-year-old woman presented to the emergency department with severe right inguinal pain. Her medical history was non-contributory and there was no known trauma or injury to the region. Amid concern for an incarcerated inguinal hernia, a computed tomography was obtained revealing a linear foreign body (FB) lateral to the femoral vessels. The FB was removed without complication at bedside and found to be a beading needle likely occultly lodged three days prior.

**Discussion:**

Occult inguinal FBs are rare but can lead to deep venous thrombosis or pulmonary embolism if in or near vessels. By nature of being occult, an absence of ingestion, insertion, or penetrative history should not preclude consideration of a FB etiology. Computed tomography imaging is crucial in determining the urgency of, and approach to, inguinal foreign body removal.

## CASE PRESENTATION

**Image 1:**
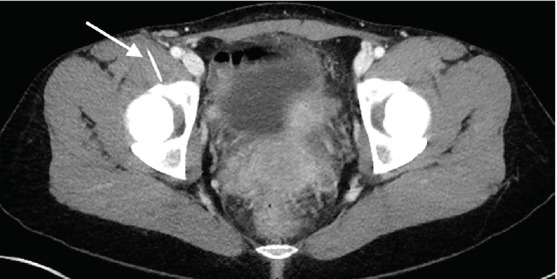
Computed tomography axial image demonstrating a radiopaque linear foreign body (arrow) passing through the iliopsoas muscle without gas or fluid along its path, resting adjacent to right anterior acetabular wall.

A 35-year-old woman presented to the emergency department with severe right inguinal pain that began while pushing a grocery cart. Physical examination revealed tenderness and fullness in the right inguinal crease without erythema, warmth, or skin trauma. Amid concern for an incarcerated inguinal hernia, a computed tomography (CT) was obtained (Image 1).

**Image 2:**
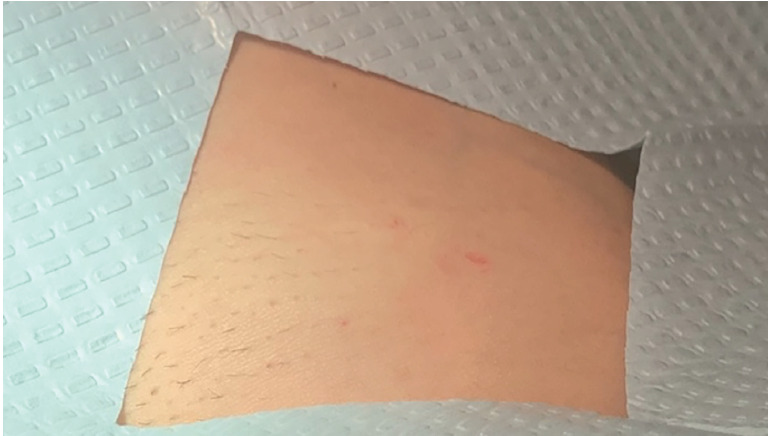
Pre-procedure image of right inguinal region demonstrating no clear puncture site for the foreign body visualized on computed tomography imaging.

The CT demonstrated a linear 3.8-centimeter (cm) foreign body (FB) extending from the skin into the right iliopsoas muscle (Image 1) without gas or fluid along its length and without vessel involvement. Upon re-interview and re-examination, the patient did not report any preceding history that would explain this finding and there was no visible point of entry on her skin (Image 2).

**Image 3:**
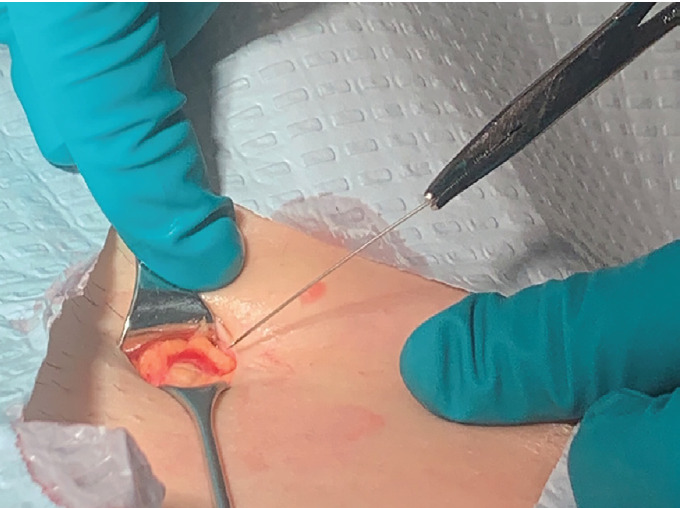
Intra-procedure image demonstrating removal of 4-centimeter beading needle.

The CT imaging confirmed this FB to be lateral to the femoral vessels in a location amenable to bedside removal in the ED. The skin was incised, and a 4-cm metallic FB was removed (Image 3).

Upon removal, the patient identified the FB as a beading needle and presumed that it had become lodged three days prior when she had fallen asleep adjacent to her beading materials. Following the FB removal, the incision was closed, and the patient was discharged without complication.

## DISCUSSION

Occult FBs are rare, although needles are common culprits due to their ease of entering tissue with minimal pain and inflammation.^1^ Inguinal needle FBs are uncommon but can generally be grouped into those associated with intravenous drug use,^2^ acupuncture needle retention,^3^ and accidental/inexplicable cases.^4^,^5^ Deep venous thrombosis may be the initial clinical manifestation of occult inguinal FBs in or near vessels.^5^ As illustrated by this case and others,^4^,^5^ FBs remain an important consideration even without known history of ingestion, insertion, or penetrative injury. Computed tomography imaging is crucial in determining the urgency of, and approach to, inguinal FB removal.

**CPC-EM Capsule**

What do we already know about this clinical entity?

Foreign body (FB) injuries are typically apparent from clinical history and have exam findings compatible with tissue injury and inflammation.

What is the major impact of the image(s)?

These images demonstrate that a FB can be present without any corresponding history or external exam findings, with the potential for serious downstream sequelae.

How might this improve emergency medicine practice?

Although rare, occult FBs must be considered by emergency providers as a source of pain despite an absence of clinical history.
